# Disruption of the AR/ZNF217/PROM2 axis sensitizes prostate cancer to ferroptosis and enzalutamide therapy

**DOI:** 10.1038/s41389-026-00649-7

**Published:** 2026-08-11

**Authors:** Wenjun Hao, Peng Liang, Wanglong Zheng, Minglu Sheng, Yulin Peng, Xiang Gao, Haolin Zhao, Yuyan Zhu, Yanlong Wang, Bo Fan, Zhihong Dai, Xu Sun, Liang Wang, Huan Qi, Ying Wang, Yan Wang, Zhenlong Yu, Zhiyu Liu

**Affiliations:** 1 Department of Urology, The Second Affiliated Hospital of Dalian Medical University, Dalian, China; 2 Liaoning Provincial Key Laboratory of Urological Digital Precision Diagnosis and Treatment, The Second Affiliated Hospital of Dalian Medical University, Dalian, China; 3 Liaoning Engineering Research Center of Integrated Precision Diagnosis and Treatment Technology for Urological Cancer, The Second Affiliated Hospital of Dalian Medical University, Dalian, China; 4 Dalian Key Laboratory of Prostate Cancer Research, The Second Affiliated Hospital of Dalian Medical University, Dalian, China; 5 Pharmaceutical Research Center, The Second Affiliated Hospital of Dalian Medical University, Dalian, China; 6 College (Institute) of Integrative Medicine, Dalian Medical University, Dalian, China; 7 Department of Urology, The First Affiliated Hospital of China Medical University, Shenyang, China; 8 Department of Pathology, The First Affiliated Hospital of Dalian Medical University, Dalian, China; 9 College of Pharmacy, Dalian Medical University, Dalian, China

**Keywords:** Prostate cancer, Cell death

## Abstract

Dysregulation of androgen receptor (AR) signaling is a key molecular driver of prostate cancer development and its transition to castration-resistant disease. Although emerging evidence indicates that AR may influence ferroptosis by modulating lipid peroxidation and antioxidant systems, the complete transcriptional network through which AR regulates ferroptosis remains incompletely defined. In this study, we identify the transcription factor ZNF217 as a direct downstream target of AR, which is aberrantly upregulated in prostate cancer and associated with poor clinical outcomes. ZNF217 enhances the transcriptional activity of its downstream effector PROM2, thereby suppressing ferroptosis and promoting cell survival. Functional assays demonstrate that ZNF217 silencing markedly amplifies ferroptotic features, whereas ZNF217 overexpression partially alleviates ferroptosis-induced cellular damage. Building on these findings, we established a drug screening system based on ZNF217 expression and identified the compound RSL3 from a panel of 13 ferroptosis inducers, which potently suppresses ZNF217 expression. Further investigations revealed that combined treatment with RSL3 and the AR antagonist enzalutamide produced a synergistic antitumor response in cellular and animal models, accompanied by marked attenuation of AR/ZNF217/PROM2 pathway activity and reduced tumor burden. Collectively, these findings identify AR/ZNF217/PROM2 signaling as a central determinant of ferroptotic vulnerability in prostate cancer and suggest that targeting this pathway may enhance sensitivity to AR inhibition, providing a molecular rationale for combination therapy in castration-resistant prostate cancer.

## Introduction

Prostate cancer represents a major global health burden, accounting for substantial cancer incidence and mortality among men worldwide [1], and its initiation and progression are critically dependent on activation of the androgen receptor (AR) signaling axis. Although androgen deprivation therapy (ADT) and second-generation AR signaling inhibitors, such as enzalutamide, can markedly prolong progression-free survival, the majority of patients ultimately experience disease progression to castration-resistant prostate cancer (CRPC), a state associated with aggressive behavior and resistance to multiple therapeutic agents [2]. Current evidence indicates that, even under castrate conditions, AR can maintain its transcriptional activity through gene amplification, enhancer activation, or the generation of constitutively active splice variants, thereby driving metabolic reprogramming and sustained tumor cell survival [3]. These observations underscore that AR is not only the predominant driver of CRPC but also a critical molecular node governing metabolic adaptation and therapeutic resistance.

Ferroptosis is an iron-driven form of regulated cell death that arises from the accumulation of lipid peroxides, in which oxidized polyunsaturated fatty acids (PUFAs) accumulate in cellular membranes and glutathione peroxidase 4 (GPX4) becomes inactivated [4]. In recent years, accumulating evidence demonstrates that ferroptosis contributes substantially to tumorigenesis, the regulation of drug sensitivity, and the orchestration of antitumor immune responses, making it a focus of intense interest in cancer therapy. Prostate cancer cells exhibit pronounced lipid metabolic reprogramming, relying heavily on fatty acid synthesis and oxidation as major energy sources, along with reinforcement of antioxidant defense systems, which collectively render these cells relatively tolerant to ferroptosis [5–8]. This metabolic phenotype suggests that alterations in lipid metabolism and redox homeostasis may be critical determinants of ferroptosis sensitivity in prostate cancer cells.

Recent studies have revealed that AR functions as a central hub linking lipid metabolism, redox balance, and iron homeostasis in prostate cancer. AR transcriptionally upregulates lipid metabolic enzymes such as acyl-CoA synthetase long-chain family member 3 (ACSL3), stearoyl-CoA desaturase 1 (SCD1), fatty acid synthase (FASN) and membrane-bound O-acyltransferase domain-containing 2 (MBOAT2), thereby increasing the enrichment of monounsaturated fatty acids (MUFAs) within cellular membrane phospholipid pools, lowering the proportion of PUFAs and suppressing lipid peroxidation; concurrently, AR represses ACSL4 expression, thereby diminishing ferroptosis susceptibility [9–11]. In addition, AR directly activates the expression of the cystine transporter subunits SLC7A11 and SLC3A2, as well as GPX4, thereby enhancing glutathione-dependent antioxidant defenses and facilitating the clearance of lipid peroxides [12–14]. Through coordinated remodeling of lipid metabolism and redox pathways, AR establishes a systemic anti-ferroptotic program that supports CRPC cells’ ability to withstand metabolic stress. This framework suggests that targeting AR signaling may disrupt ferroptosis tolerance and provide a mechanistic basis for metabolism-based combination therapies in CRPC.

The present study demonstrates that the AR/ZNF217/PROM2 axis plays a critical role in ferroptosis tolerance in CRPC. Our data show that ZNF217, an oncogenic zinc finger transcription factor, promotes CRPC resistance to ferroptosis by transcriptionally activating prominin-2 (PROM2) and thereby modulating iron homeostasis and lipid peroxidation. Mechanistically, AR directly upregulates ZNF217 expression, which, in turn, sustains PROM2-mediated antioxidant defenses and iron efflux, thereby restraining ferroptotic cell death. Functionally, ZNF217 inhibition markedly sensitizes CRPC cells to RSL3-induced ferroptosis. Furthermore, combination therapy involving RSL3, identified through ZNF217 expression–based screening, and the AR inhibitor enzalutamide synergistically suppresses the AR/ZNF217/PROM2 axis and effectively inhibits tumor growth in both cellular and animal models. Collectively, our study uncovers an AR-dependent mechanism underlying ferroptosis tolerance and highlights the AR/ZNF217/PROM2 axis as a promising entry point for ferroptosis-oriented combination strategies in castration-resistant prostate cancer.

## Materials and methods

### Data sources and preprocessing

The multi-omics and transcriptomic data used in this study were obtained from public repositories, including The Cancer Genome Atlas (TCGA), Gene Expression Omnibus (GEO), and the cBioPortal for Cancer Genomics [15]. RNA-sequencing data (STAR-Counts format) and corresponding clinical annotations for the TCGA-PRAD cohort (499 prostate cancer and 52 normal prostate samples) were downloaded from the GDC Data Portal (https://portal.gdc.cancer.gov). Raw count matrices were transformed into TPM values using gene lengths from Ensembl GRCh38, followed by log₂(TPM + 1) transformation for downstream analyses including correlation, GSEA, and survival modeling. Clinical follow-up information, including overall survival (OS), disease-specific survival (DSS), and progression-free survival (PFS), was used for prognostic analyses. GEO datasets GSE48403, GSE198488, GSE94767, GSE107299, GSE70769 and GSE70768 were downloaded and employed for differential expression and survival analyses according to their original platforms. In addition, RNA-seq data from the SU2C/PCF Dream Team project (PNAS, 2019) and the German DKFZ cohort (Cancer Cell, 2018) were obtained through cBioPortal and incorporated into subsequent analyses. All analyses were conducted in R (version 4.1.3).

### Single-gene set enrichment analysis (GSEA)

Single-gene GSEA based on ZNF217 expression was performed in the TCGA-PRAD and DKFZ prostate cancer cohorts. Samples were stratified into ZNF217 high- and low-expression groups according to the mean ZNF217 expression. GSEA software (v4.3.0) was used to assess pathway enrichment. Gene sets were obtained from the MSigDB database (v2024.1), including KEGG pathways (c2.cp.kegg_legacy) and Gene Ontology Biological Process terms (c5.go.bp). The number of permutations was set to 1000, and all other parameters were kept at default settings. Normalized enrichment score (NES), nominal *P* value, and FDR *q*-value were reported by the software, and enrichment significance was evaluated primarily based on nominal *P* < 0.05.

### Cell lines and patient specimens

Human prostate cancer cell lines (22Rv1, C4-2B, LNCaP, DU145, and PC3), the normal prostate epithelial cell line RWPE-1, and the human embryonic kidney cell line HEK293T were obtained from Hunan Fenghui Biotechnology Co., Ltd. (Changsha, China). All cell lines were authenticated by short tandem repeat (STR) profiling and were routinely tested for mycoplasma contamination using PCR-based assays (Beyotime, China) at monthly intervals to ensure identity and quality. Experiments were performed using cells within 10–20 passages after thawing. 22Rv1, C4-2B, LNCaP, PC3, and RWPE-1 cells were cultured in RPMI-1640 medium (Zhongsheng AoBang Biotechnology, Beijing, China), whereas DU145 and HEK293T cells were maintained in high-glucose DMEM (Zhongsheng AoBang Biotechnology). All media were supplemented with 10% fetal bovine serum (FBS; Gibco, USA) and 1% penicillin/streptomycin solution (Meilunbio, Dalian, China). Cells were cultured at 37 °C in a humidified incubator with 5% CO₂. When indicated, androgen-deprivation conditions were established by culturing cells in phenol red-free medium supplemented with 10% charcoal-stripped fetal bovine serum (CS-FBS; Gibco, USA). Prostate tumor tissues and paraffin-embedded specimens were obtained from the Second Affiliated Hospital of Dalian Medical University. All procedures involving human samples were approved by the Institutional Review Board of the Second Affiliated Hospital of Dalian Medical University (Approval ID: KY2024-022-01). Written informed consent was obtained from all participants, or a waiver was granted by the ethics committee when appropriate.

### Reagents and antibodies

Enzalutamide (HY-70002), RSL3 (HY-100218A), Cycloheximide (HY-12320), diallyl trisulfide (HY-117235), altretamine (HY-B0181), cisplatin (HY-17394), buthionine sulfoximine (HY-106376), Ferrostatin-1 (HY-100579), Necrostatin-1 (HY-15760) and Z-VAD-FMK (HY-16658B) were purchased from MedChemExpress (MCE, USA). Erastin (E742046), sulfasalazine (S838221), sorafenib (S828599), 6-gingerol (G810517), dihydroartemisinin (D831931), FIN56 (F872872), artesunate (A800614), and flubendazole (F834545) were obtained from Macklin (Shanghai, China). Dihydrotestosterone (DHT) was purchased from GLPBIO (USA). All small-molecule compounds were dissolved in DMSO to prepare 20 mM stock solutions, except for buthionine sulfoximine, which was dissolved in H₂O according to its solubility specifications, stored at −20 °C, and protected from light when applicable (e.g., RSL3, erastin). Working concentrations were freshly prepared by diluting stock solutions into culture medium. An anti-MBOAT2 antibody (ab121453), anti-GPX4 antibody (ab125066), anti-AR antibody (ab108341), and an anti-ZNF217 antibody (ab124927) were purchased from Abcam (Cambridge, UK), and the anti-PROM2 antibody (102-13560) was obtained from RayBiotech. All primary antibodies were used according to the manufacturer’s recommendations.

### Real-time quantitative PCR (RT-qPCR)

Total RNA was extracted from cells using TRIzol reagent (Invitrogen, USA) according to the manufacturer’s instructions. RNA purity and concentration were assessed spectrophotometrically, and 1 μg of RNA was reverse-transcribed into cDNA using the MonScript™ RTII All-in-One Mix (Monad, China). Quantitative PCR was performed using MonAmp™ SYBR Green qPCR Mix (Monad) on a QuantStudio™ 6 Flex real-time PCR system (Applied Biosystems, USA). A melt-curve analysis was conducted to confirm amplification specificity. Relative transcript levels were determined by the 2⁻ΔΔCt approach, normalized to β-actin. All measurements were performed in triplicate. Primer sequences were as follows: β-actin, forward 5′-CATCCGCAAAGACCTGTACG-3′ and reverse 5′-CCTGCTTGCTGATCCACATC-3′; AR, forward 5′-TTGGACAGTACCAGGGACCA-3′ and reverse 5′-GAGAGCTCCGTAGTGACAGC-3′; ZNF217, forward 5′-GATGTTACTCCTCCTCCGGATG-3′ and reverse 5′-CACACTTGGCCTGTATCTGCA-3′; PROM2, forward 5′-GCTCAGGAACCCAAACCTGT-3′ and reverse 5′-GGCAGGCCATACATCCTTCT-3′.

### Western blotting

Total cellular protein was extracted using RIPA lysis buffer (Beyotime, China) supplemented with protease inhibitors. Protein concentrations were quantified by the BCA method. Equivalent protein samples were resolved by 10% SDS–PAGE and electrotransferred onto PVDF membranes at 100 V for 90 min. Membranes were blocked with 5% non-fat milk in TBST for 2 h at room temperature, followed by overnight incubation at 4 °C with primary antibodies at manufacturer-recommended dilutions. After washing, membranes were incubated with HRP-conjugated secondary antibodies at room temperature for 1 h. Signals were visualized using an ECL chemiluminescence reagent (Bio-Rad, USA), imaged, and band intensities quantified with ImageJ software. β-actin was used as the loading control.

### Plasmid construction and cell transfection

To achieve stable overexpression of target genes in prostate cancer cells, cDNA sequences of the genes of interest were cloned into the PCDH lentiviral vector. Lentiviral particles were produced by co-transfecting PCDH constructs with the packaging plasmids psPAX2 and pMD2.G into HEK293T cells using PEI (Polysciences, USA). Viral supernatants were collected, filtered, and used to infect 22Rv1 cells, followed by puromycin (0.5 μg/mL) selection to establish stable overexpression cell lines. Gene knockdown was performed using siRNA transfection with Lipofectamine 2000 (Invitrogen, USA) according to the manufacturer’s instructions. Cells were harvested 48 h after transfection, and knockdown or overexpression efficiency was verified at the mRNA and protein levels by RT-qPCR and Western blotting, respectively, with β-actin as the internal control. All siRNAs were synthesized by Sangon Biotech (Shanghai, China). Two independent siRNAs targeting AR and ZNF217, as well as a negative control, were used. The sequences were as follows: si-AR-1#, Sense: 5′-GGAGCUCUCACAUGUGGAATT-3′ and Antisense: 5′-UUCCACAUGUGAGAGCUCCTT-3′; si-AR-2#, Sense: 5′-GCACCUCUCUCAAGAGUUUTT-3′ and Antisense: 5′-AAACUCUUGAGAGAGGUGCTT-3′; si-ZNF217-1#, Sense: 5′-GCCAAUUACAGAAGAGGCUAUTT-3′ and Antisense: 5′-AUAGCCUCUUCUGUAAUUGGCTT-3′; si-ZNF217-2#, Sense: 5′-CCGGCAAUUUCUUUGAGUAAATT-3′ and Antisense: 5′-UUUACUCAAAGAAAUUGCCGGTT-3′;negative control, Sense: 5′-UUCUCCGAACGUGUCACGUTT-3′ and Antisense: 5′-ACGUGACACGUUCGGAGAATT-3′.

### Colony formation assay

Colony formation assays were performed to evaluate the clonogenic capacity of prostate cancer cells. After the indicated treatments or transfections, cells were seeded into six-well plates at appropriate densities and cultured for 10–14 days until visible colonies formed. Colonies were fixed with a methanol/acetic acid (1:1) for 20 min and stained with 0.1% crystal violet. Colonies containing ≥50 cells were counted as positive using ImageJ. Results are presented as the mean ± standard deviation (SD) from three independent experiments.

### Cell viability assay

Cell viability was determined using the CCK-8 assay (Meilunbio, Dalian, China). Cells were plated in 96-well plates at optimized densities (approximately 5000–8000 cells per well) and exposed to the indicated treatments for 24–72 h, as specified for each experiment. Following treatment, CCK-8 reagent (10 μL per well) was added, and plates were incubated at 37 °C for 2 h. Absorbance at 450 nm was measured using a microplate reader (Bio-Rad iMark, USA). Dose–response curves were generated, and half-maximal inhibitory concentrations (IC₅₀) and combination indices (CI) for drug combinations were calculated using GraphPad Prism 9 and CompuSyn software based on the Chou–Talalay method.

### Transmission electron microscopy (TEM)

To observe the effects of ZNF217 knockdown on mitochondrial ultrastructure in 22Rv1 prostate cancer cells, cells were cultured under standard conditions, transfected with siRNAs as described above, and harvested at the indicated time points. Cells were fixed, dehydrated through graded ethanol, embedded in resin, and sectioned into ultrathin slices (~70 nm). Sections were stained with heavy metal solutions and examined using a transmission electron microscope (JEOL JEM-1400Plus, Japan) to evaluate mitochondrial morphology, focusing on changes in size, membrane density and cristae structure. Mitochondrial morphology was assessed qualitatively to confirm the presence of these ferroptotic features; no quantitative scoring, mitochondrial counting or blinded assessment was performed. Representative images were recorded for analysis.

### BODIPY 581/591 C11 staining for lipid peroxidation

Lipid peroxidation was assessed using the BODIPY 581/591 C11 fluorescent probe (MCE, USA). After the indicated treatments, cells were washed with PBS and incubated with 5 μM BODIPY 581/591 C11 working solution (stock dissolved in DMSO per manufacturer’s instructions) in the dark at 37 °C for 30 min. Cells were then washed with PBS, and fluorescence signals were acquired by a flow cytometer (BD FACSCanto II, BD Biosciences, USA), with excitation at 488 nm and detection through FITC and PE channels. A standard gating strategy (FSC/SSC to exclude debris, singlet gating, and live-cell selection) was applied. Flow cytometry data were acquired with sufficient event counts to ensure statistical robustness. Data were analyzed using FlowJo v10 software. Lipid ROS levels were quantified as the relative mean fluorescence intensity (MFI), normalized to the corresponding control group and expressed as fold change.

### Measurement of intracellular Fe^2+^ levels

Intracellular labile ferrous iron was quantified using a colorimetric detection kit (Elabscience, Wuhan, China). Following the indicated treatments, cells were lysed, and the resulting supernatants were reacted with the detection reagents at 37 °C for 10 min to allow color development. Absorbance was measured at 593 nm using a microplate reader (Bio-Rad iMark, USA). Fe²⁺ levels were normalized to the corresponding control group and are presented as relative Fe²⁺ levels. Data are presented as mean ± SD.

### Measurement of malondialdehyde (MDA) levels

Cellular malondialdehyde (MDA) levels were measured using a commercial TBA-based assay kit (Elabscience, Wuhan, China) according to the manufacturer’s protocol. Briefly, no fewer than 3 × 10⁶ treated cells were collected, lysed in 0.5 mL extraction buffer, and homogenized to obtain a uniform suspension. Total protein concentrations were quantified using a BCA assay (Beyotime, China). For each reaction, 0.1 mL of the sample lysate was mixed with 1 mL of the working reagent, sealed, and incubated in a 100 °C water bath for 40 min, followed by cooling and centrifugation. The supernatant (0.25 mL) was transferred to a 96-well plate, and absorbance was measured at 532 nm using a microplate reader (Bio-Rad iMark, USA). MDA levels were normalized to total protein content and expressed relative to the corresponding control group.

### Dual-luciferase reporter assay

Dual-luciferase reporter assays were performed to assess the transcriptional activities of ZNF217 and PROM2 promoters. HEK293T cells were seeded into 96-well plates and transfected with pCDH-based dual-luciferase reporter plasmids using PEI. All reporter constructs contained Firefly and Renilla luciferases on the same plasmid backbone; therefore, the total plasmid amount applied per well was 100 ng. For ZNF217, a series of 5′ promoter fragments upstream of the transcription start site (TSS) were cloned into the dual-luciferase reporter vector, including −1500 bp, −1200 bp, −1000 bp, −700 bp, and −400 bp regions. In addition, a site-directed mutant reporter plasmid targeting the predicted androgen-response element (ARE) within the ZNF217 promoter was generated to evaluate AR-dependent transcriptional regulation. For PROM2, promoter fragments spanning −500 bp and −100 bp upstream of the TSS and extending into the 5′UTR to include the predicted ZNF217-binding site were cloned to define the core regulatory region. All constructs, including the ZNF217 mutant promoter, were verified by Sanger sequencing. After 48 h of transfection, cells were lysed, and Firefly and Renilla luciferase signals were detected using a dual-luciferase reporter assay system (Quantobio, China) according to the manufacturer’s protocol. Because Firefly and Renilla luciferases were expressed from the same plasmid, Renilla activity served as an internal control for normalization. Promoter activity was quantified as the ratio of Firefly to Renilla luciferase signals.

### Chromatin immunoprecipitation followed by qPCR (ChIP-qPCR)

Chromatin immunoprecipitation followed by qPCR (ChIP-qPCR) was performed to examine AR binding to the ZNF217 promoter region in prostate cancer cells using a ChIP assay kit (Beyotime, China). Briefly, cells were cross-linked with formaldehyde, lysed, and sonicated to shear chromatin into 200–800 bp fragments. Chromatin shearing was performed using a Bioruptor Plus sonicator (Diagenode) at 20 W for 15 cycles (10 s ON/20 s OFF). After pre-clearing with Protein A + G agarose/salmon sperm DNA, chromatin was incubated with an AR antibody (Abcam, ab108341) or normal IgG control to form immune complexes, which were captured with Protein A + G agarose. Immune complexes were washed sequentially with low-salt, high-salt, LiCl, and TE buffers, eluted, reverse-crosslinked, and treated with proteinase K. DNA was purified by phenol–chloroform extraction and ethanol precipitation, and resuspended in RNase-free water. ChIP-qPCR was performed using SYBR Green Master Mix, with input DNA serving as the reference for total chromatin and IgG serving as a negative control. Primers targeting the ZNF217 promoter region were designed to span the AR-binding peak identified by ChIP-seq (forward: 5′-AGTAGATTCTTGGAACCTAGGCT-3′; reverse: 5′-CGGGTTAGAAGGCAGCTGT- 3′). Enrichment of target fragments was calculated using the ΔCt method and normalized to the IgG group, and results are expressed as mean ± SD. Statistical analyses were performed in GraphPad Prism 9.

### Immunohistochemistry (IHC)

Paraffin-embedded tissue sections (4 μm) were deparaffinized in xylene and rehydrated through a graded ethanol series. Antigen retrieval was performed by microwave treatment in EDTA buffer (pH 9.0). Endogenous peroxidase activity was blocked, and nonspecific binding sites were blocked with 5% bovine serum albumin (BSA). Sections were incubated with primary antibodies (diluted according to the manufacturer’s recommendations) at 4 °C overnight, followed by incubation with HRP-conjugated secondary antibodies (goat anti-mouse/rabbit IgG polymer) at 37 °C. Immunoreactivity was visualized with DAB, and nuclei were counterstained with hematoxylin. Sections were dehydrated through graded ethanol, cleared in xylene, and mounted. Staining was examined under a light microscope, and brown-yellow cytoplasmic or nuclear precipitates were recorded as positive signals for the target proteins.

### Mouse xenograft model

A 22Rv1 prostate cancer xenograft model was established in 8-week-old male NYG immunodeficient mice (Changsheng Biotechnology, Liaoning, China). All animal procedures were approved by the Animal Ethics Committee of Dalian Medical University and were conducted in accordance with institutional guidelines. 22Rv1 cells were digested with trypsin, washed with PBS and counted, then mixed 1:1 (v/v) with Matrigel and subcutaneously injected at approximately 5 × 10⁶ cells per mouse. Mice were monitored daily, and tumor volumes were measured every two days using calipers (volume = length × width²/2). When the average tumor volume reached ~80 mm³, mice were randomly assigned to treatment groups using a randomization procedure (*n* = 6 per group) to receive vehicle, enzalutamide (25 mg/kg, oral gavage every other day), RSL3 (8 mg/kg, intraperitoneal injection every other day), or the combination of both drugs. All drugs were dissolved in a vehicle consisting of 2% DMSO, 40% PEG-300, 5% Tween-80, and 53% normal saline. Treatments were administered for 21 days, and body weight and tumor growth were recorded throughout the experiment. Tumor measurements and endpoint assessments were performed using predefined criteria and without exclusion of animals from the final analysis. Investigators were not blinded to group allocation during treatment administration, tumor measurement, or outcome assessment. At the end of the study, mice were euthanized, and tumors were excised, weighed, photographed, and processed for paraffin embedding, IHC, and Western blot analyses.

### Statistical analysis

Sample sizes were selected based on previous studies and our prior experience with similar experimental models. For animal experiments, group sizes were determined with consideration of previous xenograft studies and ethical principles aimed at minimizing animal use while maintaining adequate statistical power. No formal statistical method or power calculation was used to predetermine sample size. No samples, animals, or data points were excluded from the analyses. All experiments were independently repeated at least three times. Data are presented as mean ± standard deviation (SD) or mean ± standard error of the mean (SEM), as indicated. Data distribution was first assessed for normality. For normally distributed data, differences between two groups were analyzed using unpaired two-sided Student’s *t*-tests, and multiple group comparisons were performed using one-way ANOVA followed by appropriate post hoc tests. For non-normally distributed data, the Wilcoxon rank-sum test was used. Multiple comparisons were adjusted using the Benjamini–Hochberg false discovery rate (FDR) method when applicable. Graphs and statistical analyses were generated using GraphPad Prism 9. A two-sided *P* value < 0.05 was considered statistically significant, with significance levels denoted as ***P < 0.001, **P < 0.01, *P < 0.05, and ns indicating no significant difference.

## Results

### ZNF217, an AR signaling target, is persistently upregulated during prostate cancer progression

**Fig. 1:**
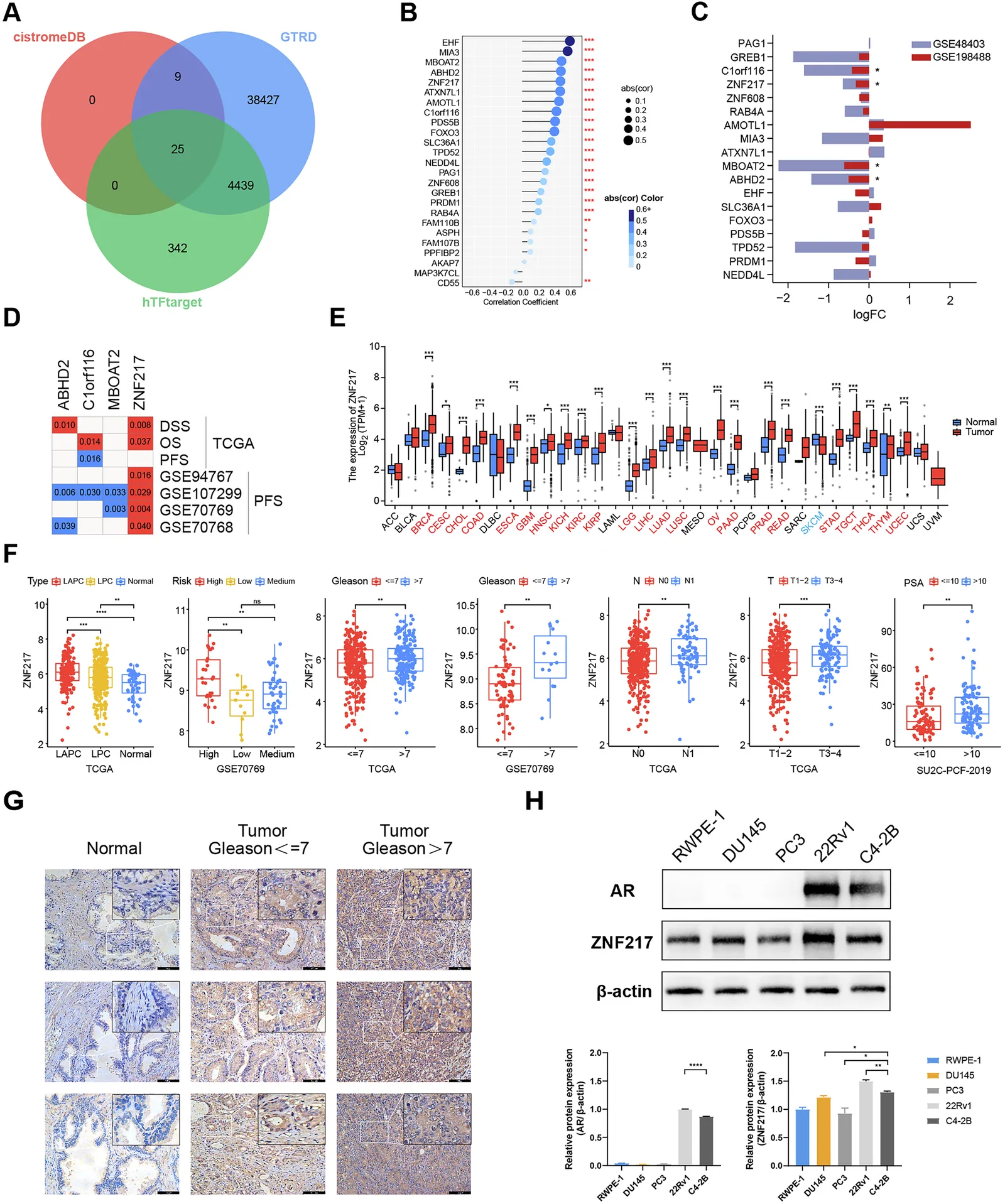
Aberrant upregulation of ZNF217 in prostate cancer correlates with poor clinical outcomes and tumor progression. **A** Overlap of AR-binding gene sets identified from the Cistrome DB, GTRD, and hTFtarget databases, yielding a set of candidate direct AR target genes. **B** Lollipop plot of Pearson correlation coefficients between AR and 25 candidate genes in the TCGA-PRAD cohort. Dot size reflects correlation strength; genes marked with “*” have *R*² > 0.2 and *P* < 0.05. **C** Bar plots showing log**₂** fold changes (logFC) of selected genes in AR-modulated transcriptomic datasets (GSE48403 and GSE198488), comparing expression before and after ADT or enzalutamide treatment. Genes labelled with “*” display concordant directions of change in both datasets with *P* < 0.05. **D** Heatmap summarizing associations between expression of candidate genes and clinical outcomes (OS, PFS, DSS) across TCGA and GEO cohorts. Colors indicate the direction of association (red, high expression associated with poor prognosis; blue, high expression associated with favorable prognosis), with *P* values from survival analyses directly annotated. **E** Landscape of ZNF217 expression across 33 human malignancies based on integrated TCGA and GTEx datasets. Boxplots compare expression in tumor (red) versus normal (blue) tissues. **F** Boxplots showing ZNF217 expression in prostate cancer patients with different clinical characteristics using integrated TCGA, GSE70769 and SU2C–PCF–2019 datasets, including disease stage (localized prostate cancer, LPC; locally advanced prostate cancer, LAPC), Gleason score, risk category, N stage, T stage and PSA levels. **G** Representative immunohistochemical staining of ZNF217 in prostate cancer specimens compared with paired non-tumorous counterparts (scale bar, 100 μm). **H** Western blot analysis of AR and ZNF217 protein expression in the normal prostate epithelial cell line RWPE-1, AR-negative prostate cancer cell lines (DU145 and PC3), and AR-positive prostate cancer cell lines (22Rv1 and C4-2B). β-actin was used as a loading control. Quantification of AR and ZNF217 protein expression normalized to β-actin is shown below. Data are presented as mean ± SD and were analyzed by Student’s *t*-test (*P < 0.05, **P < 0.01, ***P < 0.001,****P < 0.0001, ns no significance).

Given sustained AR pathway activity is a hallmark of castration-resistant prostate cancer and a major driver of therapeutic failure, we conducted an integrated multi-omics analysis to systematically identify AR-responsive genes with potential roles in disease progression. By integrating publicly available AR ChIP-seq targets from the Cistrome DB, GTRD and hTFtarget databases [16–18], we compiled a panel of 25 putative AR-binding genes (Fig. 1A). Correlation analysis in the TCGA-PRAD RNA-seq cohort revealed that AR expression exhibited a positive association with 19 candidate genes according to the established thresholds (*R*² > 0.2, *P* < 0.05; Fig. 1B). To further refine AR-responsive genes associated with therapy modulation, we interrogated two AR-intervention datasets (GSE48403 and GSE198488). Differential expression analysis revealed that C1orf116, ZNF217, ABHD2, and MBOAT2 were consistently upregulated under AR-activating conditions and downregulated upon AR pathway inhibition (Fig. 1C).

We next evaluated the clinical relevance of these candidates. Across multiple independent cohorts (TCGA-PRAD, GSE94767, GSE107299, GSE70769 and GSE70768) consistently demonstrated that elevated ZNF217 expression corresponded to unfavorable clinical outcomes, including reduced overall survival (OS), progression-free survival (PFS) and disease-specific survival (DSS) (Fig. 1D), supporting ZNF217 as a clinically meaningful AR-regulated gene. Using integrated TCGA and GTEx datasets, pan-cancer profiling revealed significant ZNF217 overexpression in approximately 70% of tumor types, including prostate adenocarcinoma (PRAD) (Fig. 1E). Within prostate cancer, ZNF217 expression increased progressively with disease stage and risk category: levels were higher in locally advanced prostate cancer (LAPC) than in localized prostate cancer (LPC), and highest in high-risk patients stratified by PSA, Gleason score and T stage. ZNF217 high expression also correlated with advanced T stage (T3–4) and lymph-node metastasis (N1). In the mCRPC SU2C/PCF Dream Team cohort, ZNF217 levels increased concurrently with rising serum PSA, further linking ZNF217expression to tumor burden (Fig. 1F).

Immunohistochemistry confirmed that ZNF217 protein expression was markedly elevated in malignant prostate samples relative to neighboring benign epithelium. Signal intensity rose with increasing Gleason score, with the strongest staining observed in tumors with Gleason score ≥7 (Fig. 1G). In line with these findings, Western blot analysis revealed maximal ZNF217 expression in AR-positive prostate cancer cell lines, including 22Rv1 and C4-2B, whereas relatively lower levels were observed in the AR-negative prostate cancer cell lines DU145 and PC3. Consistently, AR protein was predominantly expressed in 22Rv1 and C4-2B cells, exhibiting a distribution pattern similar to that of ZNF217 and suggesting a potential association between AR and ZNF217 expression (Fig. 1H). Collectively, multi-omics integration, clinical cohort analyses and experimental validation indicate ZNF217 as a key downstream effector of AR signaling that is persistently upregulated during prostate cancer progression and linked to more aggressive clinicopathological features, increased tumor load and unfavorable patient outcomes.

### AR directly binds to and transcriptionally activates ZNF217

To determine whether AR exerts its oncogenic effects in part through ZNF217, we first investigated the regulation of ZNF217 by AR signaling. In AR-positive prostate cancer cell lines (22Rv1, C4-2B, and LNCaP), culture under charcoal-stripped serum (CS-FBS) conditions significantly reduced ZNF217 protein expression compared with regular FBS conditions. DHT treatment restored ZNF217 expression, whereas this effect was effectively blocked by the AR antagonist enzalutamide (ENZ) (Fig. S1A–C). Furthermore, ectopic expression of AR in AR-negative PC3 cells markedly increased ZNF217 protein levels (Fig. S1D). Collectively, these findings indicate that ZNF217 expression is positively regulated by AR signaling.

**Fig. 2:**
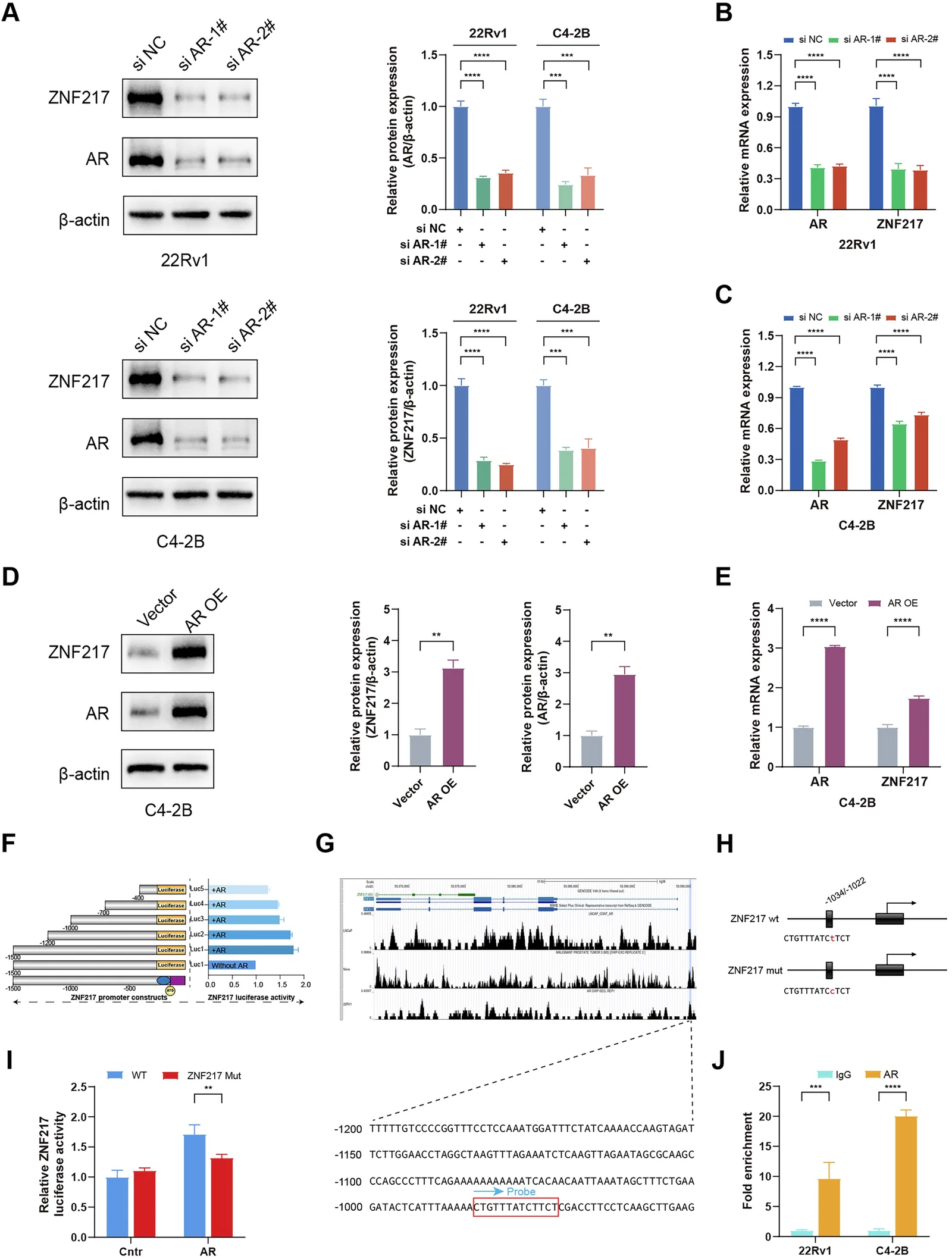
AR directly regulates ZNF217 transcriptional activity. **A** Western blot assessment of AR and ZNF217 expression following AR knockdown in 22Rv1 and C4-2B cells; the right panel shows densitometric quantification. Quantitative PCR measurement of AR and ZNF217 transcripts in 22Rv1 (**B**) and C4-2B (**C**) cells following siRNA-mediated AR silencing. Western blot (**D**) and quantitative PCR (**E**) assessment of AR and ZNF217 following AR overexpression in C4-2B cells; protein quantification is shown to the right of the blots. **F** Promoter-reporter luciferase analysis of ZNF217 in HEK293T cells under AR overexpression conditions, using constructs spanning −0 to −400, −700, −1000, −1200 and −1500 bp relative to the transcription start site (Luc1–Luc5). Relative luciferase activity was normalized to Renilla control. **G** Genome browser tracks of public AR ChIP-seq/ChIP-exo datasets from Cistrome DB showing AR occupancy at the ZNF217 promoter. Tracks include DHT-treated (10 nM) LNCaP cells (GSM2058893), 22Rv1 prostate cancer cells (GSM2537212), and prostate cancer patient specimens (GSM1071293). Light blue vertical lines indicate a prominent AR-binding region located approximately 1000–1200 bp upstream of the ZNF217 transcription start site. The lower panel shows the nucleotide sequence of this region and the predicted AR-binding motif. **H** Schematic of ZNF217 promoter luciferase constructs with wild-type (wt) or mutated (mut) AR-binding motifs. **I** Luciferase assay comparing AR-induced activation of wild-type (ZNF217-wt) versus motif-mutated (ZNF217-mut) ZNF217 promoter reporters in HEK293T cells. **J** ChIP-qPCR analysis of AR enrichment at the ZNF217 promoter region in 22Rv1 and C4-2B cells, with IgG serving as negative control. Data are presented as mean ± SD and were analyzed using Student’s *t*-test (*P < 0.05, **P < 0.01, ***P < 0.001, ****P < 0.0001).

To further validate the regulatory role of AR in ZNF217 expression, AR knockdown in 22Rv1 and C4-2B cells significantly reduced ZNF217 mRNA and protein levels, whereas AR overexpression in 22Rv1 cells markedly increased ZNF217 expression (Fig. 2A–E), indicating a positive regulatory relationship.

We then assessed whether AR directly activates ZNF217 transcription through promoter binding. A series of luciferase reporter constructs containing different lengths of the ZNF217 promoter was generated. Co-transfection with AR significantly increased ZNF217 promoter activity, with the strongest induction observed for the −1000 to −1200 bp upstream fragment (Fig. 2F), suggesting the presence of a critical AR-responsive element within this region. Notably, in the luciferase reporter system containing the 1200-bp promoter fragment, AR overexpression alone markedly increased promoter activity, whereas additional DHT treatment produced only a marginal effect (Fig. S1E), further supporting the presence of sufficient endogenous androgenic activity in regular FBS to activate AR signaling. Consistent with this, integrated analysis of public AR ChIP-seq datasets revealed prominent AR binding peaks in a promoter-proximal region (approximately −1000 to −1200 bp relative to the ZNF217 TSS) across multiple prostate cancer models, including 22Rv1, LNCaP and patient samples (Fig. 2G). Motif prediction identified a high-confidence AR-binding sequence (CTGTTTATCTTCT) within this segment (Fig. 2G).

Site-directed mutagenesis of this predicted AR-binding motif almost completely abrogated AR-induced luciferase activity, whereas the wild-type promoter remained strongly AR-responsive (Fig. 2H–I), indicating that this site is essential for AR-mediated transcriptional activation of ZNF217. Consistently, ChIP-qPCR demonstrated robust AR enrichment at this promoter region in 22Rv1 and C4-2B cells, with >8-fold enrichment relative to IgG control (*P* < 0.001; Fig. 2J). Collectively, these findings place ZNF217 under direct AR-mediated transcriptional control in prostate cancer cells.

### ZNF217 promotes prostate cancer cell proliferation and suppresses ferroptosis

**Fig. 3:**
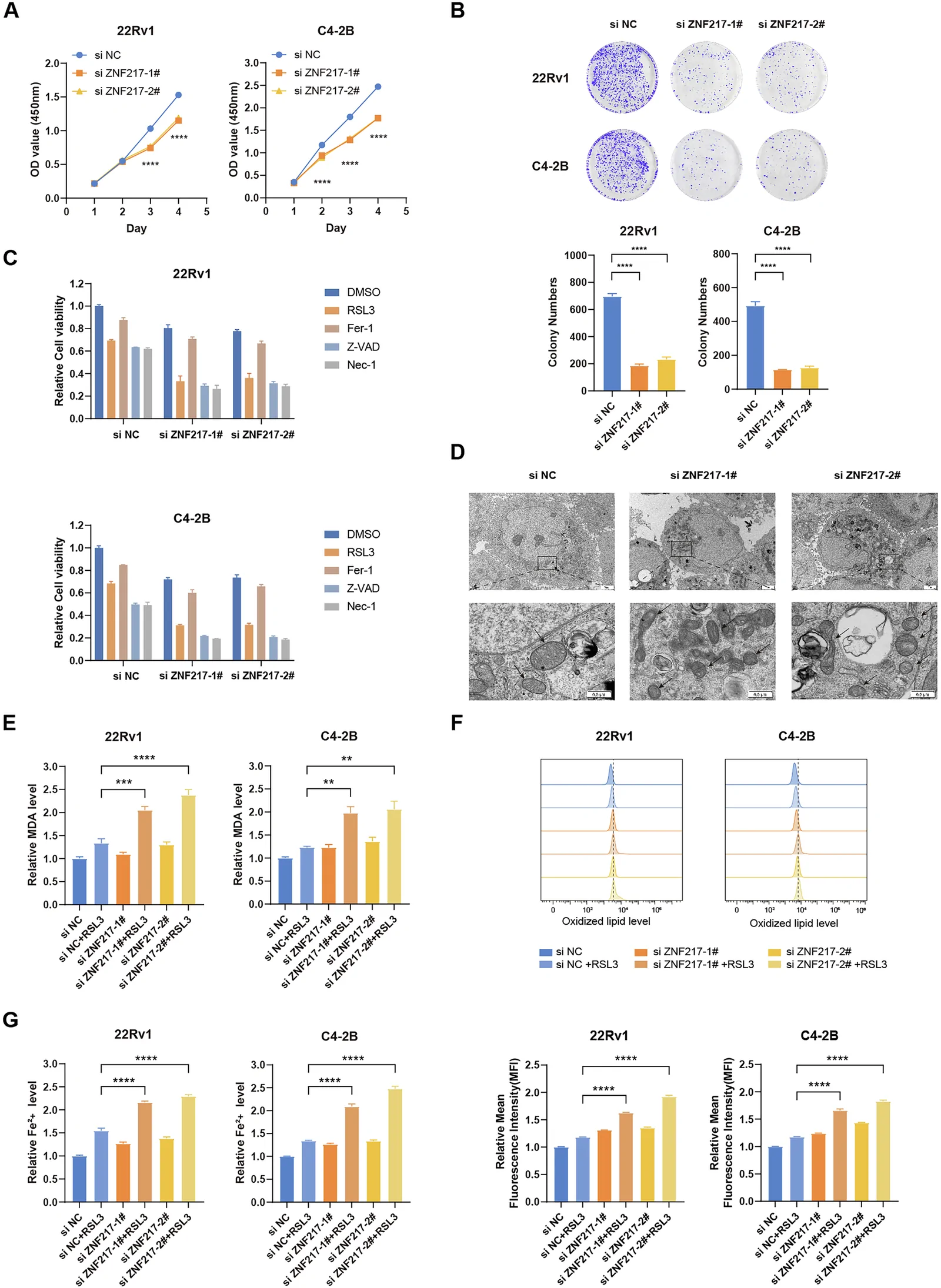
ZNF217 promotes prostate cancer cell proliferation and suppresses ferroptosis. **A** Cell proliferation assessed by CCK-8 assay in 22Rv1 and C4-2B cells following transfection with siNC or ZNF217-targeting siRNAs (siZNF217-1#, siZNF217-2#) over a 4-day period. **B** Colony formation assays assessing clonogenic growth of 22Rv1 and C4-2B cells under the indicated ZNF217 knockdown conditions; representative crystal violet-stained plates (top) and quantification of colony numbers (bottom) are shown. **C** Cell viability was evaluated by CCK-8 assay in 22Rv1 and C4-2B cells following transfection with siZNF217-1#, siZNF217-2#, or control siRNA. Cells were subsequently treated separately with RSL3 (0.5 μM), Ferrostatin-1 (Fer-1, 1 μM), Z-VAD-FMK (Z-VAD, 5 μM), or Necrostatin-1 (Nec-1, 5 μM). **D** Transmission electron microscopy (TEM) images showing mitochondrial ultrastructure in 22Rv1 cells after ZNF217 knockdown; the lower panels show enlarged views highlighting ferroptosis-associated mitochondrial alterations (scale bar, 0.5 μm). **E** Intracellular relative malondialdehyde (MDA) levels in 22Rv1 and C4-2B cells after ZNF217 knockdown and RSL3 treatment. **F** Flow cytometric analysis of lipid peroxidation using the C11-BODIPY 581/591 probe in 22Rv1 and C4-2B cells under the indicated conditions; relative mean fluorescence intensity (MFI) is shown. **G** Quantification of relative intracellular labile Fe²⁺ levels in 22Rv1 and C4-2B cells following ZNF217 knockdown and RSL3 exposure. Data are presented as mean ± SD and were analyzed by Student’s *t*-test (*P < 0.05, **P < 0.01, ***P < 0.001,****P < 0.0001).

We next assessed the functional contribution of ZNF217 in prostate cancer cells. Knockdown of ZNF217 in 22Rv1 and C4-2B cells significantly reduced colony-forming capacity and CCK-8–determined cell viability (Fig. 3A, B), whereas stable overexpression of ZNF217 in 22Rv1 cells increased both colony formation and cell viability (Fig. S2C, D). These findings indicate that ZNF217 enhances the proliferative capacity of prostate cancer cells.

To explore the underlying molecular mechanisms, we performed single-gene GSEA using ZNF217 expression as a stratification variable in the TCGA-PRAD and cBioPortal DKFZ cohorts. High ZNF217 expression was significantly enriched for gene sets related to prostate cancer, AR signaling, oxidative stress, and reactive oxygen species (ROS) responses (Fig. S2A, B), suggesting a role in redox regulation [19]. To distinguish the mode of cell death underlying the reduced cell viability observed upon ZNF217 depletion, ZNF217-knockout cells treated with the ferroptosis inducer RSL3 were co-treated with the ferroptosis inhibitor ferrostatin-1 (Fer-1), the pan-caspase apoptosis inhibitor Z-VAD-FMK (Z-VAD), or the necroptosis inhibitor necrostatin-1 (Nec-1). Notably, only Fer-1 significantly rescued the viability of ZNF217-deficient cells, whereas neither Z-VAD nor Nec-1 exerted a protective effect (Fig. 3C), indicating that cell death induced by ZNF217 loss is predominantly dependent on the ferroptotic pathway. To further validate the association between ZNF217 and ferroptosis, transmission electron microscopy was performed in ZNF217-knockdown prostate cancer cells. ZNF217 depletion induced mitochondrial alterations characteristic of ferroptosis, manifested by mitochondrial shrinkage, increased membrane density, and loss of cristae (Fig. 3D). Upon treatment with the ferroptosis inducer RSL3, ZNF217-depleted 22Rv1 and C4-2B cells exhibited markedly reduced viability compared with control cells. This increased sensitivity was accompanied by significant elevations in intracellular ferrous iron (Fe^2+^), malondialdehyde (MDA) and lipid ROS levels, as measured by biochemical assays and C11-BODIPY 581/591 flow cytometry (Fig. 3E–G). Conversely, ZNF217 overexpression conferred resistance to RSL3 challenge and blunted the accumulation of Fe²⁺, MDA, and lipid ROS (Fig. S2E–H). To explore the relationship between ZNF217 and previously reported ferroptosis regulators, we further examined the expression of GPX4 and MBOAT2 following ZNF217 knockdown. While GPX4 protein levels remained largely unchanged, MBOAT2 expression was markedly reduced upon ZNF217 depletion (Fig. S2I). Collectively, our data indicate that ZNF217 supports prostate cancer cell growth while limiting susceptibility to ferroptotic stress, and that elevated ZNF217 expression is associated with a ferroptosis-refractory phenotype.

### ZNF217 transcriptionally activates PROM2 to suppress ferroptosis in prostate cancer cells

**Fig. 4:**
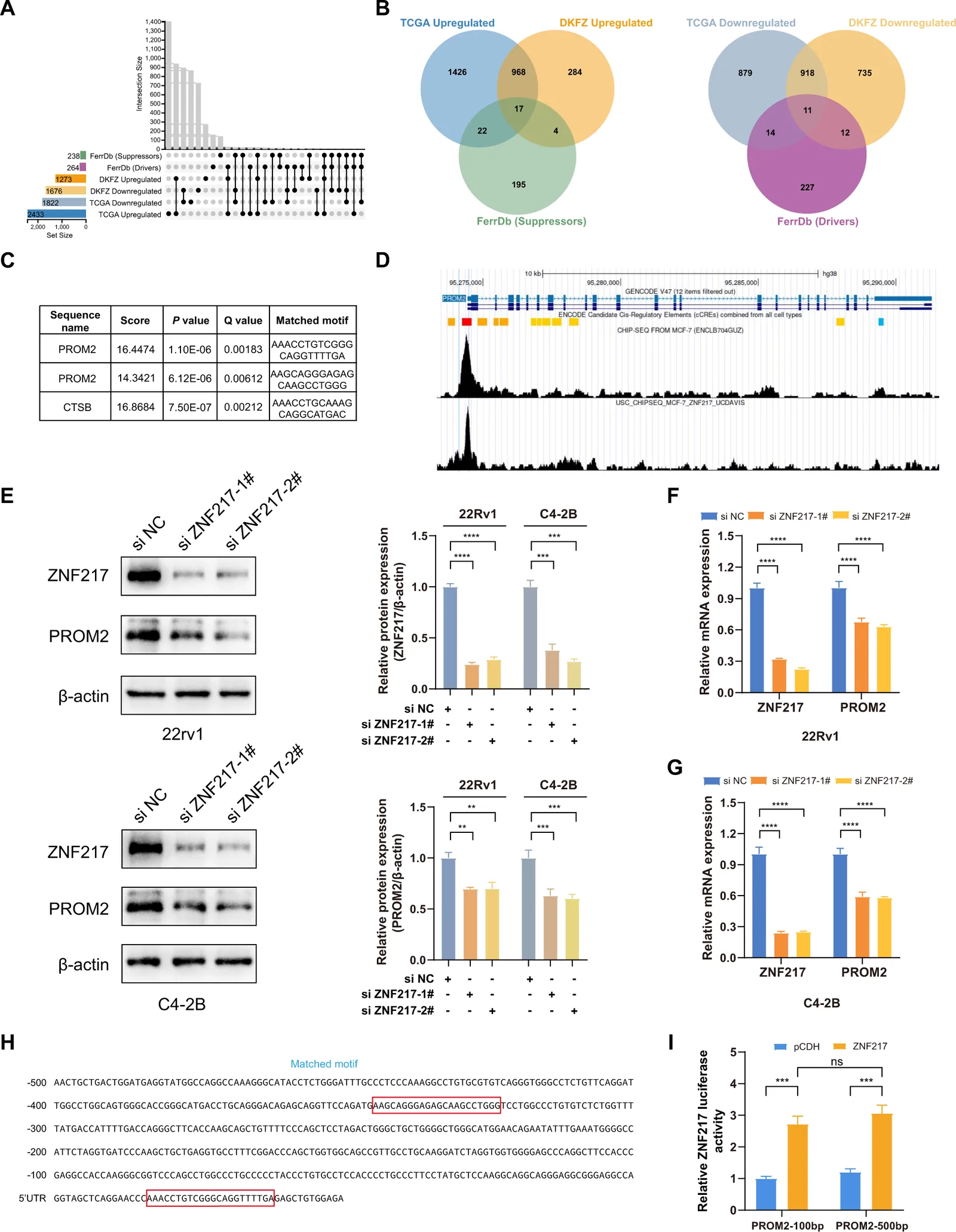
ZNF217 transcriptionally activates the ferroptosis suppressor PROM2. **A** UpSet plot illustrating the intersections of differentially expressed gene sets from the TCGA, DKFZ, and FerrDb datasets. The horizontal axis represents dataset intersections, the vertical axis indicates intersection sizes, and the left bar plot summarizes gene counts for each dataset. **B** Venn diagrams illustrate the intersections between differentially expressed genes from the TCGA and DKFZ cohorts and ferroptosis-related gene sets curated in FerrDb, including suppressors (left) and drivers (right). **C** Predicted downstream ZNF217 targets based on hTFtarget, including scores, significance levels, and consensus binding motifs. **D** Publicly available ZNF217 ChIP-seq signal tracks obtained from Cistrome DB showing ZNF217 occupancy at the PROM2 promoter region. The upper track corresponds to the ENCODE dataset GSM2827461 (ZNF217 ChIP-seq in MCF-7 cells), and the lower track corresponds to the independent USC/Farnham dataset GSM935563 (ZNF217 ChIP-seq in MCF-7 cells). The light blue shaded region indicates the predicted ZNF217-binding motif identified in (**C**). **E** Protein expression of ZNF217 and PROM2 was examined by Western blotting in 22Rv1 and C4-2B cells following control or ZNF217-targeted siRNA treatment; densitometric quantification is shown to the right. qPCR analysis of ZNF217 and PROM2 mRNA levels in 22Rv1 (**F**) and C4-2B (**G**) cells after ZNF217 knockdown. **H** Schematic of the PROM2 promoter region showing the predicted ZNF217-binding motifs. **I** Dual-luciferase reporter assays using constructs containing different lengths of the PROM2 promoter co-transfected with ZNF217 expression vector, demonstrating the effect of ZNF217 on PROM2 promoter activity. Data are presented as mean ± SD and analyzed using Student’s *t*-test (*P < 0.05, **P < 0.01, ***P < 0.001, ns no significance).

To delineate the downstream effectors through which ZNF217 modulates ferroptosis, we performed differential expression analysis in the TCGA and DKFZ prostate cancer cohorts, stratified by ZNF217 expression (|log₂FC| ≥ 1.5, *P* < 0.05). Candidate genes were intersected with ferroptosis drivers and suppressors curated in FerrDb (Fig. 4A) [20]. In ZNF217-high samples, 17 upregulated genes overlapped with ferroptosis suppressors and 11 downregulated genes overlapped with ferroptosis drivers (Fig. 4B), suggesting that ZNF217 may facilitate ferroptosis resistance by coordinately upregulating ferroptosis suppressors and downregulating ferroptosis drivers. We then used hTFtarget to predict ZNF217 binding confidence across these candidates. PROM2 showed the highest predicted ZNF217 binding score among upregulated genes, whereas CTSB was the top among downregulated genes (Fig. 4C). Cistrome ChIP-seq data revealed a strong ZNF217 binding peak in the PROM2 promoter region, but no obvious enrichment at the CTSB promoter (Figs. 4D and S3A), implicating PROM2 as a potential direct ZNF217 transcriptional target.

Functional validation in 22Rv1 and C4-2B cells showed that ZNF217 knockdown significantly reduced PROM2 mRNA and protein levels, while ZNF217 overexpression markedly increased PROM2 expression (Figs. 4E–G and S3B, C), indicating positive regulation. Luciferase reporter assays using PROM2 promoter fragments demonstrated that ZNF217 significantly enhanced the activity of constructs containing the −100 bp and −500 bp promoter regions, with no significant difference between these two fragments (Fig. 4H, I). This pattern suggests that ZNF217 binds within the proximal promoter region of PROM2 to drive transcription. Taken together, these data indicate that ZNF217 engages the PROM2 regulatory region to enhance its transcription, thereby facilitating a ferroptosis-resistant phenotype in prostate cancer cells.

To further validate the functional dependency of PROM2 in ZNF217-mediated regulation of ferroptosis, a PROM2-knockout model was established in prostate cancer 22Rv1 cells (Fig. S4A). On this basis, ZNF217 siRNA was transfected into control cells (sgCtrl) and PROM2-knockout cells (sgPROM2), followed by assessment of cell viability and ferroptosis-related phenotypes. CCK-8–based measurements showed a marked reduction in cell viability following ZNF217 depletion in the sgCtrl background; however, in sgPROM2 cells, further depletion of ZNF217 did not result in an additional decrease in cell viability (Fig. S4B). Consistently, in sgCtrl cells, ZNF217 knockdown markedly enhanced Fe²⁺ accumulation, lipid peroxidation, and malondialdehyde (MDA) production, whereas in sgPROM2 cells, these ferroptosis-associated parameters remained unchanged following ZNF217 knockdown (Fig. S4C–E). Taken together, the absence of PROM2 abrogated the effects of ZNF217 depletion on cell viability and ferroptosis-associated features, supporting a PROM2-dependent mechanism for ZNF217-mediated ferroptosis regulation in prostate cancer cells.

### RSL3 inhibits ZNF217 transcription to enhance the antitumor efficacy of enzalutamide

**Fig. 5:**
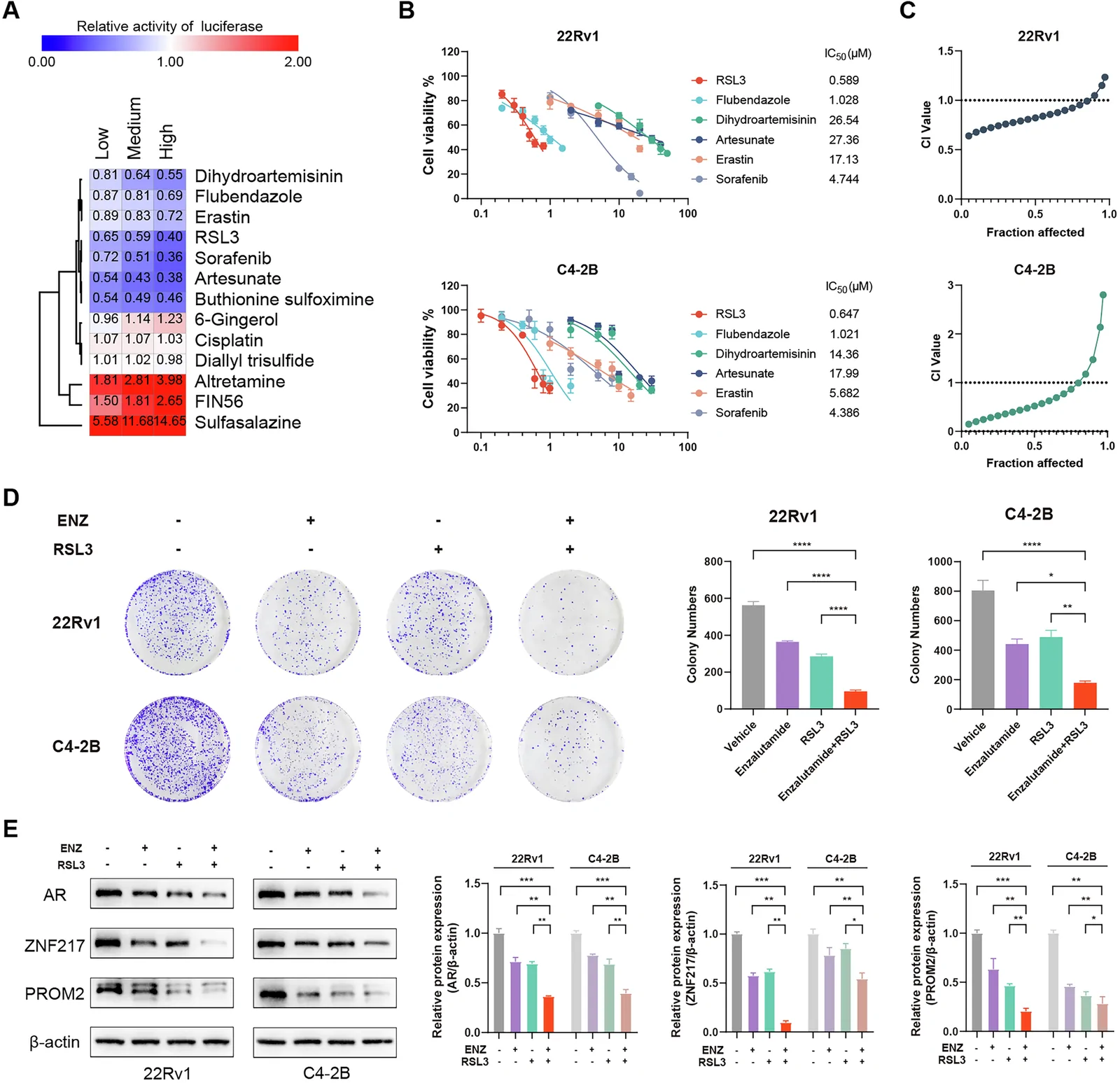
RSL3 synergizes with enzalutamide to inhibit prostate cancer cell growth and downregulate the AR/ZNF217/PROM2 axis. **A** Screening of 13 ferroptosis-related small-molecule inducers using a ZNF217 promoter-driven luciferase reporter system. 22Rv1 cells were treated for 48 h with low-, medium- and high-concentration settings for each compound, and relative luciferase activity was measured. The heatmap shows relative promoter activity across drugs and doses, with colors ranging from blue (low activity) to red (high activity). **B** Dose–response curves for RSL3, flubendazole, dihydroartemisinin, artesunate, erastin and sorafenib in 22Rv1 and C4-2B cells, with corresponding IC₅₀ values. **C** Chou–Talalay combination index (CI) analysis of RSL3 and enzalutamide (ENZ) in 22Rv1 and C4-2B cells. The x-axis indicates the fraction affected (Fa) and the y-axis shows CI values for different dose combinations. CI < 1 indicates synergy. **D** Colony-forming ability of 22Rv1 and C4-2B cells following treatment with vehicle, RSL3, ENZ, or their combination for the indicated duration. Representative crystal violet–stained images are shown on the left, with corresponding colony quantification on the right. **E** Protein expression of AR, ZNF217, and PROM2 in 22Rv1 and C4-2B cells following ENZ and/or RSL3 treatment was examined by Western blotting, with β-actin as the loading control and densitometric analysis presented on the right. Data are presented as mean ± SD and analyzed using one-way ANOVA followed by Tukey’s post hoc test (*P < 0.05, **P < 0.01, ***P < 0.001, ****P < 0.0001).

To identify pharmacologic modulators of ZNF217 transcription, we established a luciferase-based screening platform driven by the ZNF217 promoter and tested 13 representative ferroptosis-inducing small molecules [21]. In this screen, only RSL3, erastin, flubendazole, dihydroartemisinin, artesunate, sorafenib and buthionine sulfoximine (BSO) significantly decreased ZNF217 promoter–driven luciferase activity within certain dose ranges, whereas other compounds had no suppressive effect and some even increased promoter activity at specific doses (Fig. 5A). Because BSO exhibited effective concentrations in the millimolar range, far above conventional experimental use, it was not pursued further in downstream assays.

We next compared the cytotoxicity of selected candidates in AR-positive prostate cancer cell lines. In both 22Rv1 and C4-2B cells, RSL3 exhibited the strongest growth-inhibitory effect, with superior potency compared with other compounds tested (Fig. 5B). Based on these data, RSL3 was selected as the primary candidate for subsequent studies. Chou–Talalay combination analysis revealed that RSL3 and the AR inhibitor enzalutamide produced combination index (CI) values < 1 across most dose combinations in both 22Rv1 and C4-2B cells, indicating synergistic growth inhibition (Fig. 5C). Consistently, colony formation assays showed that combined RSL3 and enzalutamide treatment markedly reduced clonogenic survival compared with either monotherapy, with concordant effects in both cell lines (Fig. 5D).

At the molecular level, Western blotting demonstrated that either RSL3 or enzalutamide alone partially decreased AR, ZNF217 and PROM2 protein levels, whereas combined treatment produced a more pronounced downregulation of all three components of the AR/ZNF217/PROM2 axis (Fig. 5E). In addition, both RSL3 and enzalutamide decreased MBOAT2 protein levels, and the combined treatment produced an even stronger inhibitory effect (Fig. S5A). This finding is consistent with previous reports showing that AR inhibition downregulates MBOAT2 and enhances ferroptosis sensitivity [10]. To further clarify the role of ZNF217 in these effects, we examined changes in PROM2 and MBOAT2 expression under ZNF217 knockdown conditions. The results showed that ZNF217 silencing significantly reduced PROM2 expression, while enzalutamide or RSL3 treatment further enhanced this inhibitory effect. In contrast, MBOAT2 expression was also markedly reduced following ZNF217 knockdown; however, enzalutamide or RSL3 treatment still further suppressed its expression (Fig. S5B). Together with previous studies identifying MBOAT2 as a key downstream effector of AR signaling, these findings suggest that MBOAT2 and PROM2 may be regulated through distinct mechanisms.

Given that RSL3 significantly suppressed ZNF217 promoter activity, we further investigated its underlying mechanism. In AR-positive prostate cancer cells, Western blot analysis showed that RSL3 treatment markedly reduced both AR and ZNF217 protein levels (Fig. 5E). To determine whether RSL3 acts through regulation of AR expression, we further examined changes in AR transcription and protein stability. RT-qPCR results showed that AR mRNA levels were not significantly altered after RSL3 treatment (Fig. S5C). However, cycloheximide (CHX) chase experiments demonstrated that RSL3 markedly accelerated AR protein degradation and shortened its half-life (Fig. S5D), indicating that RSL3 primarily attenuates AR signaling by reducing AR protein stability rather than by suppressing its transcription. This may represent a key mechanism underlying its inhibitory effect on ZNF217 expression.

Notably, in the AR-negative PC3 cell line, RSL3 treatment also reduced ZNF217 protein expression (Fig. S5E), suggesting that the inhibitory effect of RSL3 on ZNF217 is not entirely dependent on AR signaling. Therefore, in addition to accelerating AR protein degradation, RSL3 may regulate ZNF217 through additional AR-independent mechanisms. Taken together, these results demonstrate that RSL3 significantly suppresses ZNF217 expression and synergizes with enzalutamide to inhibit the growth of AR-positive prostate cancer cells, leading to more effective downregulation of the AR/ZNF217/PROM2 signaling axis.

### RSL3 enhances the antitumor effect of enzalutamide in vivo via AR/ZNF217/PROM2 axis

**Fig. 6:**
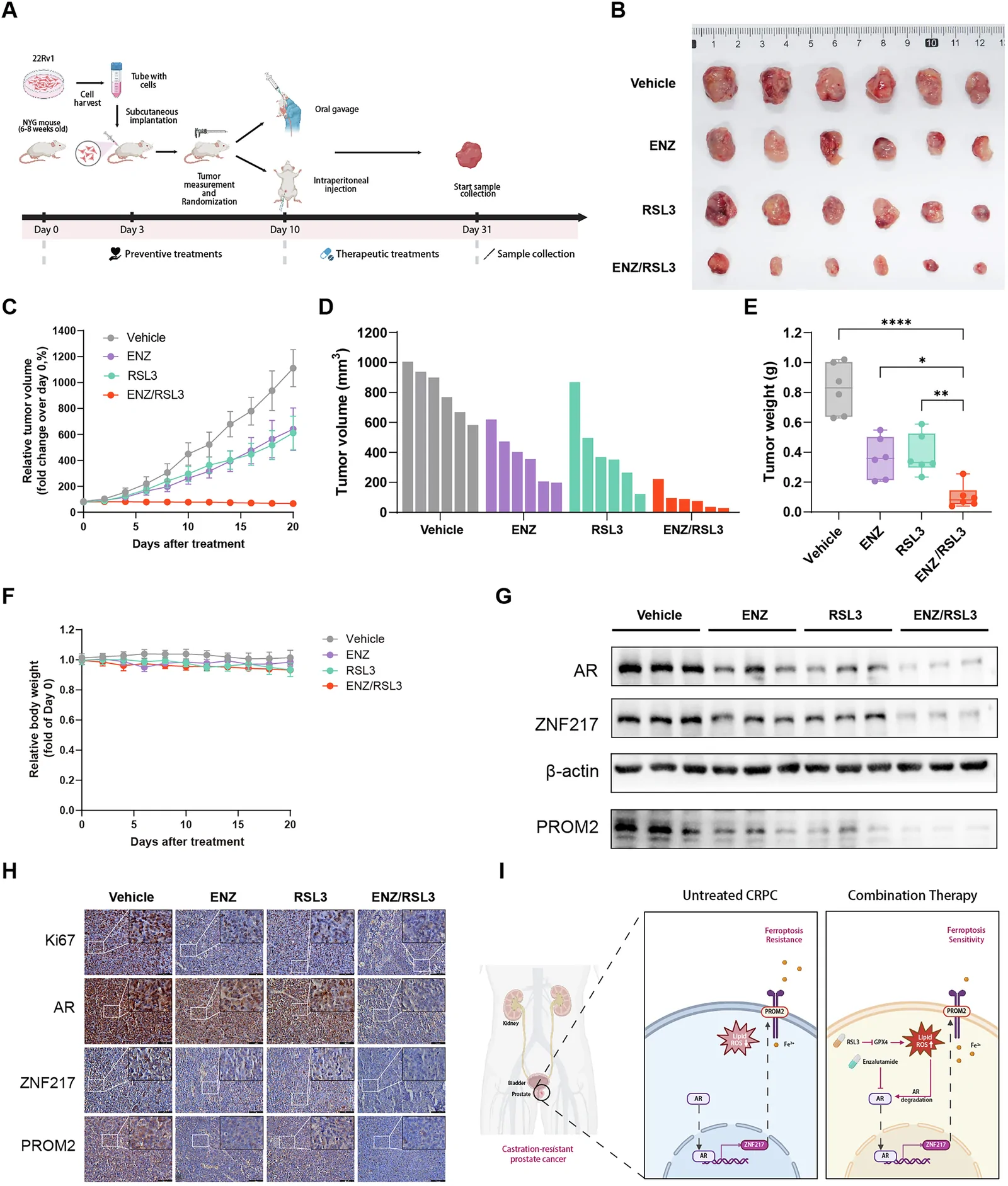
Enzalutamide plus RSL3 synergistically suppresses prostate cancer growth in vivo and downregulates the AR/ZNF217/PROM2 axis. **A** Schematic of the in vivo experimental design. Male NYG immunodeficient mice were injected subcutaneously with 22Rv1 cells and, after tumor establishment, were randomized into four groups: vehicle, enzalutamide (ENZ), RSL3, and ENZ/RSL3 combination. ENZ was administered by oral gavage and RSL3 by intraperitoneal injection as indicated in the treatment schedule. **B** Representative images of excised tumors from each treatment group at the experimental endpoint. **C** Tumor growth curves showing relative tumor volume (fold change relative to baseline at treatment initiation) over time for each treatment group, normalized to tumor size at treatment initiation. Data are presented as mean ± SD. **D** Distribution of endpoint tumor volumes for individual mice in each group. **E** Boxplots summarizing endpoint tumor weights in each treatment group. Statistical analysis was performed using one-way ANOVA followed by Tukey’s post hoc test (*P < 0.05, **P < 0.01, ***P < 0.001, ****P < 0.0001). **F** Longitudinal monitoring of relative body weight in NYG immunodeficient mice receiving Vehicle, enzalutamide (ENZ), RSL3, or combination therapy (ENZ/RSL3). Body weight was normalized to each mouse’s baseline (Day 0) prior to treatment initiation. Data are presented as mean ± standard deviation (mean ± SD). **G** Western blot analysis of AR, ZNF217 and PROM2 protein levels in tumor tissues from each group, with β-actin as loading control. **H** Representative immunohistochemistry images of Ki-67, AR, ZNF217 and PROM2 staining in paraffin-embedded tumor sections from the four treatment groups. **I** Schematic model summarizing the mechanism by which enzalutamide combined with the ferroptosis inducer RSL3 suppresses CRPC progression.

To assess the antitumor activity of RSL3 and its combinatorial effect with enzalutamide, a subcutaneous 22Rv1 xenograft model was generated in male NYG immunodeficient mice and initiated treatment once tumors reached a predefined volume (Fig. 6A). Mice were randomized into four groups: vehicle, enzalutamide, RSL3, or the combination. Both enzalutamide and RSL3 monotherapy significantly slowed tumor growth compared with vehicle, whereas the combination group exhibited the greatest tumor suppression (Fig. 6C). Tumor volume trajectories showed markedly delayed growth in the combination group, and endpoint tumor weights were substantially lower than in either monotherapy group, with partial tumor regression observed in some mice (Fig. 6B, D, E), consistent with a synergistic anti-tumor effect. To assess the in vivo toxicity of each treatment regimen, we continuously monitored the body weight of mice in each group during the experimental period. The results showed that no obvious decreasing trend in body weight was observed in any group throughout the treatment course (Fig. 6F).

Immunohistochemical analysis demonstrated a marked decrease in Ki-67 staining in tumors receiving the combination treatment relative to either monotherapy or vehicle control, indicating more effective suppression of tumor cell proliferation (Fig. 6H). In parallel, AR, ZNF217, and PROM2 staining intensities were further decreased in the combination group compared with either enzalutamide or RSL3 alone. Western blot analysis of tumor lysates corroborated these findings, showing the lowest levels of AR, ZNF217, and PROM2 in the combination arm (Fig. 6G, H). These data demonstrate that RSL3 and enzalutamide cooperate in vivo to inhibit prostate cancer growth, at least in part by jointly downregulating the AR/ZNF217/PROM2 axis and contributing to ferroptosis-associated anti-tumor responses (Fig. 6I).

## Discussion

Castration-resistant prostate cancer (CRPC) is a clinically advanced form of the disease, marked by rapid tumor progression and the emergence of treatment resistance. Although second-generation AR antagonists such as enzalutamide substantially delay disease progression, most patients ultimately develop acquired resistance and relapse [22]. Increasing evidence indicates that CRPC cells survive under endocrine therapy by engaging multilayered metabolic reprogramming and antioxidant defenses, thereby attenuating therapy-induced stress [23, 24]. Ferroptosis is a distinct mode of regulated cell death that relies on iron-dependent lipid peroxidation and has been increasingly implicated in the interplay between tumor metabolic reprogramming and therapeutic sensitivity [25, 26]. Previous studies indicate that AR influences the expression of a broad set of genes governing iron homeostasis and antioxidant defense, thereby modulating ferroptosis sensitivity [9–14]. However, whether AR also shapes ferroptosis via previously unrecognized downstream transcriptional networks remains unclear. In this study, we identify and characterize an AR/ZNF217/PROM2 signaling axis that contributes to ferroptosis tolerance in CRPC. Our findings reveal an AR/ZNF217/PROM2 regulatory axis in which AR transcriptionally activates ZNF217, which in turn enhances PROM2 expression to limit ferroptotic vulnerability in prostate cancer cells. This axis provides mechanistic insight into how AR-dependent transcriptional programs support ferroptosis resistance and offers a rationale for combinatorial therapeutic strategies in CRPC.

ZNF217 is a well-recognized oncogenic transcription factor that drives tumor cell proliferation, maintains cancer stem–like features, and contributes to therapeutic resistance across multiple cancer types, with elevated expression frequently linked to adverse clinical outcomes [27–30]. Our data extend these observations by implicating ZNF217 as a critical mediator of AR-dependent ferroptosis regulation in CRPC. Mechanistically, we show that AR directly binds to and activates the ZNF217 promoter, thereby increasing ZNF217 transcription in prostate cancer cells. Notably, during validation of AR responsiveness, we observed that ZNF217 expression under standard FBS culture conditions differed only modestly from that observed following DHT stimulation. Previous studies have reported that conventional FBS contains detectable levels of endogenous androgens, whereas even low concentrations of DHT are sufficient to activate AR signaling [31, 32]. Therefore, endogenous androgens present in standard FBS may be sufficient to partially maintain basal AR activity under routine culture conditions. This interpretation is consistent with our observation that androgen depletion using CS-FBS reduced ZNF217 expression, whereas supplementation with exogenous DHT restored its expression, further supporting positive regulation of ZNF217 by androgen/AR signaling. ZNF217 shows elevated expression in prostate cancer specimens and CRPC-derived cell models, with higher levels associated with adverse clinical features and unfavorable survival outcomes. Functionally, ZNF217 overexpression attenuates RSL3-induced ferroptotic cell death, whereas ZNF217 knockdown leads to typical ferroptotic ultrastructural changes, including reduced mitochondrial volume, increased mitochondrial membrane density and diminished cristae, and enhances sensitivity to ferroptosis induction. Notably, MBOAT2 has recently been identified as a GPX4-independent ferroptosis regulatory pathway. Unlike the canonical GPX4 antioxidant system, MBOAT2 primarily modulates ferroptosis susceptibility by regulating membrane phospholipid fatty acid composition and lipid remodeling rather than directly participating in GPX4-mediated peroxide detoxification [10]. In this context, we further observed that ZNF217 knockdown did not significantly alter GPX4 protein expression but was accompanied by a reduction in MBOAT2 levels. These findings suggest that the ferroptosis-regulatory function of ZNF217 may not primarily depend on the classical GPX4–GSH antioxidant axis. Instead, ZNF217 may influence ferroptosis through GPX4-independent regulatory mechanisms involving molecules such as MBOAT2. Therefore, the role of ZNF217 in ferroptosis regulation may extend beyond conventional antioxidant pathways and involve broader modulation of GPX4-independent ferroptosis networks. Together, these findings support a model in which ZNF217 functions to inhibit ferroptosis downstream of AR signaling and serves as a central node connecting AR-driven transcriptional programs to ferroptosis resistance in CRPC.

Through integrative multi-omics analyses, we further identified PROM2 as a key downstream target of ZNF217 and subsequently demonstrated that ZNF217 directly binds to and transcriptionally activates the PROM2 promoter, thereby enhancing PROM2 expression. Notably, under PROM2-deficient conditions, the reduction in cell viability and the enhancement of ferroptosis-related phenotypes induced by ZNF217 silencing were largely abolished, indicating that the ferroptosis-regulatory function of ZNF217 is functionally dependent on the presence of PROM2. PROM2 has been recognized as an important regulator of iron homeostasis that promotes the trafficking and release of ferritin-containing vesicles through multivesicular bodies and exosomes, thereby reducing the intracellular labile iron pool and limiting the initiation of iron-dependent lipid peroxidation [33]. In addition to PROM2-mediated regulation of iron homeostasis, accumulating evidence suggests that ZNF217 itself may participate in other aspects of iron metabolism. Previous studies have reported that ZNF217 can modulate intracellular iron accumulation by suppressing ferroportin-mediated iron efflux, implying a potential role in the regulation of transmembrane iron export [34]. Taken together with previous findings, our results suggest that the role of ZNF217 in iron homeostasis may extend beyond a single iron metabolic process and instead involve coordinated regulation of multiple iron homeostasis modules. Among these, transcriptional activation of PROM2 and the subsequent modulation of iron availability appear to represent a major mechanism through which ZNF217 suppresses ferroptosis, whereas its reported association with ferroportin-mediated iron export may constitute an additional regulatory layer that remains to be further elucidated. Collectively, these findings indicate that ZNF217 not only regulates iron homeostasis through transcriptional activation of PROM2, but may also integrate multiple iron metabolism–related processes, thereby playing a pivotal role in the development of ferroptosis resistance in CRPC.

At the pharmacological level, our small-molecule screening based on a ZNF217 promoter-driven luciferase reporter system identified RSL3 as a potent suppressor of ZNF217 transcriptional activity. RSL3 not only markedly reduced ZNF217 promoter activity but also exhibited pronounced growth-inhibitory effects in AR-positive prostate cancer cells. Mechanistically, RSL3 treatment significantly decreased AR protein levels without affecting AR mRNA expression. Furthermore, cycloheximide (CHX) chase assays demonstrated that RSL3 accelerated AR protein degradation and shortened its half-life, suggesting that the inhibitory effect of RSL3 on AR signaling primarily occurs at the level of protein stability. Given that AR was identified in this study as a direct transcriptional activator of ZNF217, the reduction in AR protein stability induced by RSL3 may represent an important mechanism underlying the suppression of ZNF217 expression. Increasing evidence suggests that ferroptosis-associated lipid peroxidation and oxidative stress not only contribute to the execution of ferroptotic cell death but also influence cellular proteostasis networks, including the ubiquitin–proteasome system (UPS) and autophagy–lysosome system (ALS), thereby promoting the degradation of specific proteins [35]. In light of our observation that the GPX4 inhibitor RSL3 significantly shortened the half-life of AR protein without altering AR mRNA expression, we speculate that lipid peroxidation and oxidative stress resulting from GPX4 inhibition may contribute to AR protein degradation, thereby attenuating AR signaling activity. In other words, ferroptosis induction may not only function at the downstream execution stage but also feed back to suppress the AR/ZNF217/PROM2 signaling axis, thereby further amplifying ferroptosis-associated antitumor effects.

Notably, RSL3 also reduced ZNF217 expression in AR-negative PC3 cells, suggesting that its regulatory effect on ZNF217 is not entirely dependent on AR signaling and may involve additional mechanisms that remain to be elucidated. More importantly, the combination of RSL3 and the AR antagonist enzalutamide exhibited marked synergistic antitumor activity both in vitro and in vivo. Combination treatment resulted in a substantial reduction in tumor volume, a marked decrease in Ki67-positive cells, and more pronounced suppression of the AR/ZNF217/PROM2 signaling axis. These findings suggest that RSL3 enhances the antitumor efficacy of enzalutamide not only through ferroptosis induction but also by attenuating AR/ZNF217/PROM2 signaling. Although enzalutamide and RSL3 act through distinct mechanisms, both ultimately converge on the AR-dependent ferroptosis regulatory network. Enzalutamide primarily blocks downstream signaling by suppressing the transcriptional activity of AR, whereas RSL3, in addition to directly inducing ferroptosis, may indirectly attenuate the activity of the AR/ZNF217/PROM2 axis through GPX4 inhibition-associated oxidative stress and the consequent reduction in AR protein stability, thereby exerting a dual-hit antitumor effect. These findings further underscore the functional importance of the AR/ZNF217/PROM2 axis in the regulation of ferroptosis in CRPC, and suggest that concurrent targeting of AR signaling and the ferroptosis pathway may hold promising therapeutic potential.

In addition, we observed that RSL3 treatment was accompanied by a reduction in MBOAT2 expression, while enzalutamide similarly decreased MBOAT2 levels, with the combination treatment resulting in a more pronounced suppression. Notably, ZNF217 silencing also decreased MBOAT2 expression; however, enzalutamide or RSL3 treatment further reduced MBOAT2 levels even in the context of ZNF217 depletion. Given that MBOAT2 has been identified as an AR-regulated GPX4-independent ferroptosis suppressor, these findings suggest that ZNF217 may contribute to the regulation of MBOAT2 expression. In other words, AR-mediated regulation of MBOAT2 may be partially dependent on ZNF217-related mechanisms while also involving additional downstream effector pathways. These results further suggest that AR regulation of ferroptosis likely depends on multiple interconnected downstream effector modules, while the ZNF217/PROM2 axis identified in this study provides a new mechanistic perspective for understanding AR-mediated ferroptosis resistance. Notably, enzalutamide resistance remains one of the most significant clinical challenges in the management of CRPC. Although the functional role of ZNF217 has not been directly evaluated in established enzalutamide-resistant models in the present study, our findings identify ZNF217 as a critical downstream effector of AR signaling that contributes to the maintenance of ferroptosis resistance. Previous studies have shown that the development of enzalutamide resistance is often accompanied not by a complete loss of AR signaling, but rather by its persistent activity through mechanisms such as AR amplification, AR splice variant expression, and ligand-independent AR activation [36]. Therefore, even in the context of enzalutamide resistance, targeting the downstream ZNF217–PROM2 axis may provide a means to bypass certain upstream resistance mechanisms and restore tumor cell sensitivity to ferroptosis induction. These observations suggest that ZNF217 is not only an important effector of AR-mediated ferroptosis regulation, but may also participate in biological processes associated with acquired resistance in CRPC. Therefore, whether ZNF217 can serve as a therapeutic target for overcoming endocrine therapy resistance warrants further investigation in enzalutamide-resistant models.

In summary, this study delineates an AR/ZNF217/PROM2 signaling axis that sustains cell survival in CRPC by suppressing ferroptosis. Directly regulated by AR, ZNF217 functions as a key oncogenic transcription factor that activates PROM2 to modulate iron handling and lipid peroxidation, thereby conferring resistance to ferroptotic stress. Disrupting this axis—particularly through combined AR pathway blockade and ferroptosis induction—effectively compromises the growth advantage of AR-dependent tumor cells and improves the response to enzalutamide treatment in preclinical models. These findings position the AR/ZNF217/PROM2 axis as a central determinant of ferroptosis tolerance in CRPC and provide a mechanistic rationale for combination strategies that concurrently inhibit AR signaling and trigger ferroptosis.

Nonetheless, it is important to acknowledge several limitations of this study. First, the mechanistic investigations were primarily conducted in AR-positive prostate cancer cell lines and subcutaneous xenograft models. The functional role of the AR/ZNF217/PROM2 axis has not yet been systematically evaluated in established enzalutamide-resistant models. In addition, the biological significance of this signaling axis in AR-low or neuroendocrine prostate cancer remains to be determined. Second, although RSL3, as a canonical GPX4 inhibitor, serves as a valuable experimental tool for investigating ferroptosis mechanisms, its pharmacokinetic properties and limitations in vivo may restrict its direct translational potential.

Future work should focus on developing drug-like inhibitors targeting ZNF217 or PROM2, optimizing combination regimens with clinically applicable ferroptosis modulators, and prospectively validating AR/ZNF217/PROM2 axis activity and its predictive value for therapy response in well-annotated patient cohorts. Overall, our study deepens the understanding of ferroptosis regulation in CRPC and suggests new avenues to overcome endocrine resistance and refine precision treatment strategies for prostate cancer.

## Conclusion

In conclusion, this study identifies the AR/ZNF217/PROM2 axis as a previously unrecognized transcriptional program that links androgen receptor signaling to ferroptosis resistance in castration-resistant prostate cancer. Disruption of this axis, achieved through combined AR inhibition and ferroptosis induction, effectively compromises tumor cell survival and enhances the antitumor efficacy of enzalutamide in preclinical models. These findings highlight the AR/ZNF217/PROM2 axis as a central determinant of ferroptosis tolerance in CRPC and provide a mechanistic rationale for integrating ferroptosis-based strategies with endocrine therapy to overcome treatment resistance.

## Supplementary information

Supplementary material

## Data Availability

Data will be made available on request.
