# Supplementary material for: Disruption of the AR/ZNF217/PROM2 axis sensitizes prostate cancer to ferroptosis and enzalutamide therapy
Source: Oncogenesis. 2026 Aug 11;15(1):58. doi: 10.1038/s41389-026-00649-7 (PMC13740269; doi:10.1038/s41389-026-00649-7)
Supplement: Supplementary file 1 [file 41389_2026_649_MOESM1_ESM.docx]

**Supplementary Figure S1. **AR signaling positively regulates ZNF217 expression in prostate cancer cells.** **A,** B, C Western blot analysis of ZNF217 protein expression in C4-2B **(A)**, 22Rv1 **(B)**, and LNCaP **(C)** cells cultured under different androgen conditions. Cells were maintained in medium supplemented with either fetal bovine serum (FBS) or charcoal-stripped fetal bovine serum (CS-FBS), followed by treatment with dihydrotestosterone (DHT) and/or enzalutamide (ENZ) as indicated. β-actin was used as a loading control. Quantification of ZNF217 protein levels normalized to β-actin is shown on the right. **D** Western blot analysis showing the effect of ectopic AR overexpression on ZNF217 expression in AR-negative PC3 cells. β-actin served as a loading control. **E** Luciferase reporter assay showing the transcriptional activity of a reporter construct containing the 1200-bp upstream promoter region of ZNF217 following AR overexpression and DHT stimulation. Relative luciferase activity was normalized to the promoter reporter + empty vector (pCDH) group.** Data are presented as mean ± SD and analyzed by Student’s t test (*P < 0.05, **P < 0.01, ***P < 0.001,****P < 0.0001).

**Supplementary Figure S2. ZNF217 overexpression promotes prostate cancer cell proliferation and attenuates ferroptosis. A** Gene set enrichment analysis (GSEA) of ZNF217 in the TCGA prostate adenocarcinoma (PRAD) cohort, showing enrichment of androgen receptor signaling, reactive oxygen species response, oxidative stress and prostate cancer-related pathways in tumors with high ZNF217 expression. **B** GSEA of ZNF217 in the DKFZ prostate cancer RNA-seq dataset from cBioPortal, confirming enrichment of similar pathways. Genes are ranked by ZNF217 expression; NES indicates normalized enrichment score and P denotes enrichment significance. **C** CCK-8 assays of C4-2B cells transfected with control vector (Vector) or ZNF217 overexpression plasmid (ZNF217 OE), monitoring proliferation over 4 days. **D** Clonogenic growth of C4-2B cells under control or ZNF217 overexpression conditions was assessed by colony formation assay; representative crystal violet staining is shown on the left, with corresponding colony counts on the right. **E** Cell viability was measured by CCK-8 assay in C4-2B cells under vector control or ZNF217 overexpression conditions following 48 h exposure to RSL3. **F** Relative MDA levels in 22Rv1 cells under the indicated conditions. **G** Relative Fe²⁺ levels in 22Rv1 cells under the indicated conditions. **H** Flow cytometric analysis of ROS fluorescence intensity in each group, measured as relative mean fluorescence intensity (MFI). **I** Western blot analysis of ZNF217, MBOAT2, and GPX4 protein expression following siRNA-mediated knockdown of ZNF217 in C4-2B cells. Data are presented as mean ± SD and analyzed by Student’s t test (*P < 0.05, **P < 0.01, ***P < 0.001,****P < 0.0001).

**Supplementary Figure S3. Effects of ZNF217 overexpression on PROM2 expression in prostate cancer cells. A** ChIP-seq signal tracks from Cistrome DB showing ZNF217 binding in the CTSB promoter region; blue shaded region indicates the motif locations corresponding to those listed in Fig. 4C. **B** ZNF217 and PROM2 protein expression in 22Rv1 cells following transfection with control vector or ZNF217 overexpression, as determined by Western blot, with densitometric quantification shown on the right. **C** ZNF217 and PROM2 mRNA expression in 22Rv1 cells following ZNF217 overexpression, as measured by qPCR. Data are presented as mean ± SD and were analyzed by Student’s t-test (*P < 0.05, **P < 0.01).

**Supplementary Figure S4. PROM2 is required for ZNF217-mediated suppression of ferroptosis. A** Validation of PROM2 knockout efficiency at the protein level in 22Rv1 cells, with β-actin used as a loading control. **B** Relative cell viability in control cells (sgCtrl) and PROM2-knockout cells (sgPROM2) following co-transfection with ZNF217 siRNA. **C** Relative intracellular Fe²⁺ levels under different treatment conditions. **D** Flow cytometric analysis of lipid peroxidation using BODIPY 581/591 C11, with relative mean fluorescence intensity (MFI) shown for each group. **E** Relative intracellular malondialdehyde (MDA) levels under different treatment conditions.Data are presented as mean ± SD and were analyzed by Student’s t-test (*P < 0.05, **P < 0.01, ***P < 0.001,ns,no significance).

**Supplementary Figure S5. RSL3 suppresses ZNF217 expression through both AR-dependent and AR-independent mechanisms. A Western blot analysis of PROM2 and MBOAT2 protein expression in C4-2B cells treated with enzalutamide (ENZ), RSL3, or their combination. β-actin served as a loading control. **B** Western blot analysis of ZNF217, PROM2, and MBOAT2 protein expression following siRNA-mediated knockdown of ZNF217 in 22Rv1 cells with or without ENZ and RSL3 treatment. β-actin served as a loading control. **C** RT-qPCR analysis of AR mRNA expression in 22Rv1 cells following RSL3 treatment. Data are presented as mean ± SD. ns, not significant. **D** Cycloheximide (CHX) chase assay showing AR protein stability in 22Rv1 cells treated with vehicle or RSL3. Cells were harvested at the indicated time points after CHX treatment, and AR protein levels were analyzed by western blotting. Quantification of relative AR protein abundance is shown on the right. **E** Western blot analysis of ZNF217 protein expression in AR-negative PC3 cells following RSL3 treatment. β-actin served as a loading control.**
